# Bacteremia after *Bacillus clausii* administration for the treatment of acute diarrhea: A case report

**DOI:** 10.7705/biomedica.5662

**Published:** 2021-10-15

**Authors:** Juan Pablo García, John Alexander Alzate, Julián Andrés Hoyos, Edilberto Cristancho

**Affiliations:** 1 Semillero de Investigación de Medicina Interna, Universidad Tecnológica de Pereira, Pereira, Colombia Universidad Tecnológica de Pereira Universidad Tecnológica de Pereira Pereira Colombia; 2 Departamento de Medicina Interna, Universidad Tecnológica de Pereira, Pereira, Colombia Universidad Tecnológica de Pereira Universidad Tecnológica de Pereira Pereira Colombia; 3 Laboratorio de Microbiología, Hospital Universitario San Jorge, Pereira, Colombia Hospital Universitario San Jorge Pereira Colombia

**Keywords:** Bacillus clausii, bacteremia, diarrhea, probiotics, gram-positive bacteria, Bacillus clausii, bacteriemia, diarrea, probióticos, bacterias Gram positivas

## Abstract

*Bacillus clausii* is a gram-positive rod used as a probiotic to treat diarrhea and the side effects of antibiotics such as pseudomembranous colitis. We report a case of *B. clausii* bacteremia in a non-immunocompromised patient with active peptic ulcer disease and acute diarrhea. The probiotic was administered during the patient's hospitalization due to diarrhea of infectious origin. *B. clausii* was identified in the bloodstream of the patient through Matrix- Assisted Laser Desorption Ionization-Time of Flight (MALDI-TOF) days after her discharge. Given the wide use of probiotics, we alert clinicians to consider this microorganism as a causative agent when signs of systemic infection, metabolic compromise, and hemodynamic instability establish after its administration and no pathogens have been identified that could explain the clinical course.

## Resumen

*Bacillus clausii* es un bacilo Gram positivo usado como probiótico para tratar la diarrea y efectos adversos de los antibióticos, como la colitis pseudomembranosa. Se reporta un caso de bacteriemia por *B. clausii* en una paciente inmunocompetente con enfermedad ulcerosa péptica activa y diarrea aguda. El probiótico le fue administrado durante la hospitalización debido al origen infeccioso de la diarrea. *B. clausii* se identificó en el torrente sanguíneo de la paciente, utilizando espectrometría de desorción/ionización mediante láser asistido por matriz (*Matrix-Assisted Laser Desorption/Ionization*, MALDI), acoplada a un detector de iones (*Time of Flight*, TOF) (MALDI-TOF), días después de haber sido dada de alta.

Dado el amplio uso de los probióticos, alertamos a los clínicos para que consideren este microorganismo como agente causal cuando se detecten signos de infección sistémica, compromiso metabólico, e inestabilidad hemodinámica tras su administración, y no se haya identificado ningún patógeno que pueda explicar el cuadro clínico.

*Bacillus clausii* is a gram-positive, spore-forming rod, widely used as a probiotic (^1^). Probiotics have been used for hundreds of years to treat different diseases. Since the 1960s they have been used to treat viral diarrhea in children and the side effects of antibiotic administration (^2^). Antibiotic-associated side effects and *Clostridioides difficile* diarrhea are well-known scenarios where probiotics are proven to be efficient (^3^). Recent studies on the safety of *B. clausii* administration have concluded that it has intrinsic resistance mechanisms to some antibiotics (e.g. macrolides), but no toxin-producing genes or transferrable antimicrobial resistance, which makes it very safe (^4^,^5^). However, other studies have shown no related side effects linked to its use (^6^-^8^), and, on the other hand, side effects have been inconsistently reported and not adequately assessed. Anyway the World Health Organization has acknowledged probiotics might be responsible for systemic infections and deleterious metabolic activity (^9^).

## Ethical considerations

The patient gave her informed consent to access and submit her clinical data as a case report for scientific purposes. We submitted the manuscript and the informed consent to the ethics committee at *Universidad Tecnológica de Pereira* and obtained its endorsement to publish the case.

**Table 1:** Blood analysis, renal function, electrolyte control, and hemostatic parameters

	Previous to admission	Admission day	Reference values	
White blood cells (cell/ml)	10,510	8,080	4,500	-10,000
Neutrophils (cell/μΙ)	8,600	6,230	2,200	- 6,500
Hemoglobin (g/dl)	13.5	14.3	13.5	- 17.5
Platelets (cell/μΙ)	198,000	240,000	150,000-	350,000
Chloride (mmol/L)	79	76	98	- 107
Potassium (mmol/L)	2.2	2	3.6	- 5
Sodium (mmol/L)	122	122	137	- 145
Glucose (mg/dl)	98	105	60	- 110
Creatinine (mg/dl)		0.3	0.66	- 1.25
Blood urea nitrogen (mg/dl)		9	9	- 21
Thromboplastin partial time (s)		30.1	23.6	- 34.8
Prothrombin time (s)		15.3	11	- 15

## Case report

An 87-year-old woman arrived at the emergency room of a third-level hospital in Colombia reporting 12 days of watery, abundant, fetid diarrhea accompanied by brown-colored vomit and melena. She was referred from a first-level hospital with several laboratory tests completed. After admission, critical care clinicians evaluated the patient and admitted her to the intermediate care unit. Her medical history included arterial hypertension, chronic obstructive lung disease (COPD), and a cholecystectomy.

The patient appeared somnolent throughout the initial physical evaluation. She also had elevated systolic blood pressure (150/80 mm Hg) and tachypnea (22 breaths per minute), she had no fever (36,1°C), her pulse was 80 beats per minute, her oxygen saturation 97%, the Glasgow Coma Scale score was 13/15, she had dried oral mucosa and rales in both of her pulmonary fields. The examination was otherwise normal.

The previous laboratory findings and those during the patient's admission are summarized in table 1. We initially administered ampicillin/sulbactam (1.5 grams every six hours) as *Klebsiella pneumoniae* was documented in a urine culture brought by the patient (taken five days before admission) and bacterial diarrhea was suspected. There was no previous or recent documentation of any urinary symptoms and, therefore, asymptomatic bacteriuria was suspected.

Two days later, an upper gastrointestinal endoscopy was done. It showed three Forrest Class III gastric ulcers for which proton pump inhibitors were administered. Sodium, potassium, and water deficits were in constant reposition. The patient did not present new episodes of melena or vomit, only occasional diarrheic depositions during her stay at the intermediate care unit.

Based on her clinical evolution, the patient was then transferred to the internal medicine unit ten days after admission. She appeared disorientated, but no additional signs or symptoms were present, aside from diarrheic depositions. An astringent diet was started but abdominal pain and diarrhea persisted. The internal medicine physician decided to prescribe an Enterogermina™ probiotic (2 billion/5 ml *B. clausii* spore suspension, strains: SIN, O/C, T, and N/R) every six hours as of the 16^th^ day of her hospital stance.

On day 19, we interviewed the patient again in search of urinary symptoms and she reported having dysuria and abdominal pain, which she associated with a previous bladder catheterization. At this point, urine analysis, Gram stain, culture, and antimicrobial susceptibility testing were done. The urine analysis revealed positive leukocyte esterase, pyuria, and bacteriuria. The Gram stain identified a Gram-negative rod characterized three days later in the urine culture as *Escherichia coli* with an inhibitor-resistance TEM (IRT) pattern as shown by evidence of resistance to ampicillin (MIC ≥32 μg/ ml), ampicillin/sulbactam (MIC ≥32 μg/ml), and preserved sensibility to all cephalosporins. We established a diagnosis of probable urinary tract infection (UTI) and, given that the antimicrobial susceptibility testing results were only available 3 days later, we started the empirical administration of cephalothin (1 g/6 h) pending results.

**Figure 1:**
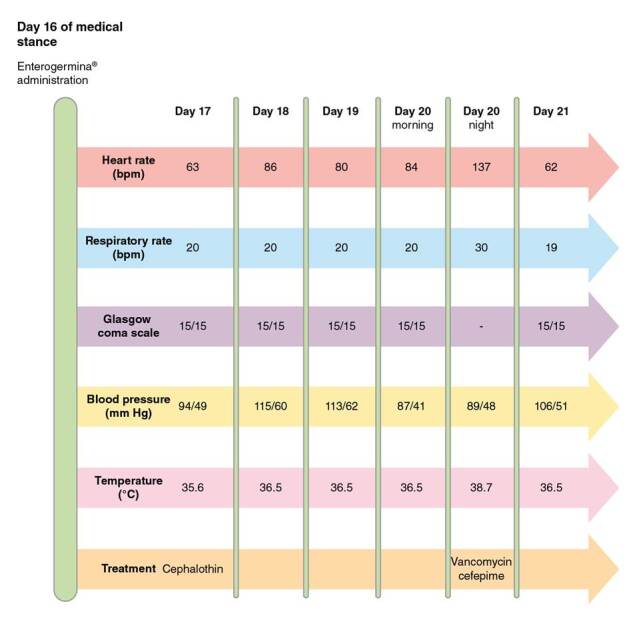
Summary of bacteremia course

On the 20^th^ day of the hospital stay, the patient began shivering, sweating, and did not appear well. Her heart rate was 137 beats per minute, her respiratory rate was 30 breaths per minute, her temperature was 38.7 °C, her blood pressure was 89/48 mm Hg, and her oxygen saturation was 88% without oxygen support. There were signs of phlebitis on the right upper limb where the peripheral catheter was placed. The blood analysis showed a white blood cell count of 9,020/mm^3^ [4,500-10,000], neutrophilia of 89%, hemoglobin of 9.8 g/dl [12-15.5], and C-reactive protein of 6.3 mg/dl [0-5] (table 2). A pair of anaerobic and aerobic blood cultures were drawn from peripheral intravenous access. Cephalothin was suspended and intravenous vancomycin (1 g/12 h) and cefepime (1 g/12 h) were started to target microorganisms responsible for sepsis in the context of a Gram-negative rod UTI and Gram-positive cocci phlebitis. Supplementary oxygen was administered by nasal cannula due to low oxygen arterial pressure. Figure 1 summarizes the clinical course after the administration of Enterogermina™.

**Table 2:** Laboratory and microbiology test results on the 20th day of hospital stance

	**20** ^th^ **day of** **hospital stance**	26^th^ day of hospital stance	Reference values
White blood cells (cell/ml)	9,020	4,960	4,500 - 10,000
Neutrophils (cell/μl)	7,810	2,460	2,200 - 6,500
Hemoglobin (g/dl)	9.8	10.9	13.5 - 17.5
Mean corpuscular volume (fl)	85.5	83.4	82 - 96
Red cell distribution width (%)	14.4	14.5	11 - 15.5
Platelets (cell/pl)	257,000	246,000	150,000 - 350,000
C-reactive protein (mg/dl)	6.3	-	0 - 5
Serum creatinine (mg/dl)	0.5	-	0.52 - 1.04
Blood urea nitrogen (mg/dl)	8	-	7 - 18
Arterial blood gases (20th day)			
pH	7.48		
pCO_2_ (mm Hg)	24		35 - 45
pO_2_ (mm Hg)	66		80 - 110
HCO_3_ std (mmol/L)	21.1		-
A-aD^3^O_2_	26		-
Potassium (mmol/L)	3		3,6 - 5
Sodium (mmol/L)	130		137 - 145
Glucose (mg/dl)	122		60 - 110
Blood cultures (20^th^ day)			
Aerobic	Gram-positive rods		None
Anaerobic	Gram-positive rods		None
Gastrointestinal Film Array Panel™ (22^nd^ day)			
Enteroaggregative and Enteropathogenic *E. coli*			

One day later, the patient's vital signs were back to normal. She tolerated oral intake, but still had diarrhea. Periodically, we assessed the renal function in creatinine and blood urea nitrogen (BUN) samples, which were never out of range. However, abdominal pain and diarrhea persisted. On day 22, we did coprology, stool culture, gastrointestinal FilmArray™, colonoscopy, and abdominal ultrasonography in search of the infectious source of diarrhea. Four days later, the FilmArray™ was positive for enteroaggregative and enteropathogenic *E. coli* (Protocol: Stool FA v2.3, Pouch: GI Panel v2.1). The other microbiological tests and diagnostic images were normal. By the 26^th^ day, a new complete blood cell count was within normal range, except for the persistence of mild anemia.

**Figure 2:**
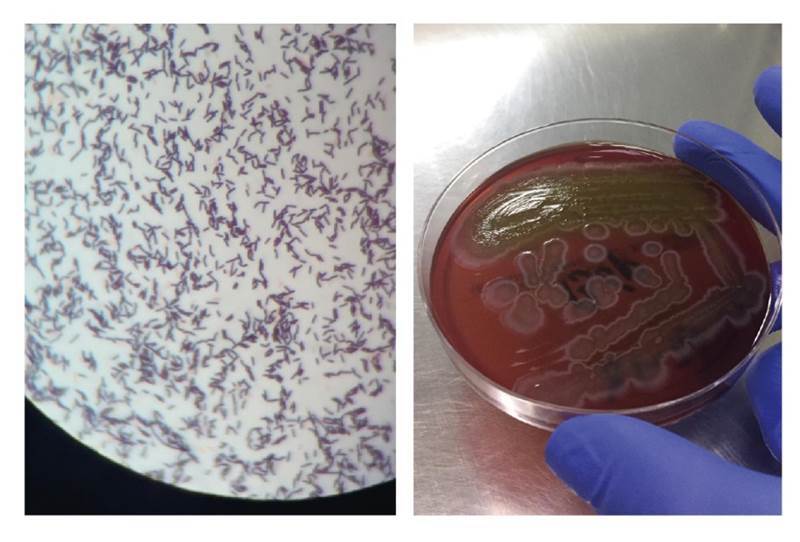
Gram stain, blood culture, and MALDI-TOF results. Left: Blood culture, Gram stain, 100X. Right: Gram-positive rod colonies that grew on blood culture in solid medium, Agar Columbia (BioMérieux brand). MALDITOF result: Type of sample: blood culture. Identified microorganism: *Bacillus clausii*. This is a sensitive and high-precision technique for the identification of microorganisms through mass spectrometry analysis. Methods sequencing the 16s subunit have a 98% concordance for microorganism identification (Score: 2.4, highly reliable identification at genera and species levels)

The results from the blood culture Gram stain were available on day 22 (figure 2), upon which we added intravenous ampicillin (2 g/4 h) to the previous antimicrobial therapy to target the Gram-positive rods identified. An infectious disease specialist assessed the therapy and decided to start intravenous gentamicin (5 m/kg in two doses per day for 7 days), continue the recently added ampicillin, and suspend the empiric therapy with vancomycin and cefepime. The patient's clinical course improved and her vital signs were within normal range. The last episode of diarrhea was on the 27^th^ day. The 7-day antimicrobial treatment was completed and supplementary oxygen was gradually removed. The patient was discharged on the 33^rd^ day with complete clinical recovery.

As the typification of the Gram-positive rod identified in the blood culture Gram stain was not possible, we resorted to MALDI-TOF spectrometry whose results were available 2 days after the patient was discharged reporting the presence of *B. clausii* (figure 2).

## Discussion

We reported the case of an 87-year-old female patient with upper gastrointestinal bleeding, diarrhea of infectious origin, and recent use of proton pump inhibitors in whom probiotics containing *B. clausii* were administered and later identified as the cause of bacteremia and hemodynamic instability. We discuss here previously published case reports of bacteremia due to *B. clausii* administration, recommended scenarios to administer probiotics, and the potential risk factors/clinical profiles that could make a patient susceptible to bacteremia caused by probiotics containing *Bacillus spp*.

The systemic infections, especially Bacillus spp. bacteremia, are rare conditions and, frequently, they are considered contaminant bacteria in blood cultures. The most common agent of Bacillus spp. bacteremia is *Bacillus cereus* but there are reports of bacteremia by probiotics containing *Bacillus subtilis* and *B. clausii* like the case we are now reporting (^10^,^11^).

Recently, three cases of immunocompromised patients who developed clinically significant bloodstream infections by *B. clausii* were reported (^12^). Two patients had stage IV lung cancer and the third one presented with septic shock due to ischemic colitis. As in these cases, our patient also had a fever and an increased count of white blood cells. While these patients died from other causes not related to *B. clausii* bacteremia, our patient survived throughout the hospital stance after adequate treatment of upper gastrointestinal bleeding and timely antimicrobial therapy. A limitation in our case report is the absence of antimicrobial susceptibility testing on *B. clausii.*

Conversely, the isolate from one of the reported patients was penicillin-, clindamycin-, and tetracycline-resistant.

Princess, *et al*. published a case report of bacteremia in an immunocompetent adult with acute diarrhea under broad-spectrum antibiotics prescribed to treat *K. pneumoniae* respiratory and surgical wound infection in a patient that had evacuating craniectomy after cerebral vein thrombosis and intraparenchymal bleeding (^13^). The patient also had a fever after probiotic administration. *B. claussi* was initially isolated in blood cultures as a Grampositive rod and identified through a MALDI-TOF assay, as was our case. We treated our patient with ampicillin plus gentamicin while Princess, et al. prescribed teicoplanin; our patient's isolate was penicillin-resistant only (MIC: 32 μg/ml) but, besides vancomycin and ciprofloxacin, no other antibiotics were tested given there were no interpretative guidelines. Another limitation of our study was that no control blood culture was done to assess the microbiological response to treatment.

Joshi, *et al*. reported the case of a pediatric patient with congenital heart disease and recurrent lower respiratory tract infections that received *B. clausii* probiotic to treat acute diarrhea (^14^) and was treated with IV vancomycin. The isolate from three blood culture samples was identified through MALDI-TOF. The strain was not associated with penicillin resistance but it is noteworthy that *B. clausii* bacteremia recurred although there was no repeated probiotic dose and the agent persisted in the blood culture despite appropriate vancomycin IV therapy.

The main conclusion from this case series is the importance of being cautious when using probiotics, especially if there is a risk of a fatal outcome. Our case report adds to a previous one of *B. clausii* bacteremia with no underlying immunocompromising conditions such as active cancer or severe malnourishment. Even though there is no formal description of risk factors to develop infections by this probiotic, we can conclude that they may occur irrespective of the patient's immune status. In table 3 we summarize the profile of the patients reported highlighting those characteristics that may behave as risk factors and encouraging awareness among attending physicians. It has yet to be determined if bacteremia by *B. clausii* could be related to worse outcomes as there are now two out of six patients who succumbed to conditions apparently not related to probiotic administration. The conditions related to an increased risk of bacteremia due to other types of probiotics (e.g., *Lactobacillus*) are older age, congenital immune deficiencies, treatment with antitumoral chemotherapy or ionizing radiation, extensive gastrointestinal ulcers, treatments with broad-spectrum antibiotics, and diabetes (^15^).

**Table 3:** Profile of patients documented with bacteremia due to Bacillus clausii administration

Authors	Year of publication	Age	Patients' profile	Treatment	Antimicrobial susceptibility
Gargar JD, *et al*.	2019	ND	Stage IV lung cancer and pneumonia	ND	One case reported resistance to clindamycin, penicillin, and tetracycline.
Gargar JD, *et al*.	2019	ND	Stage IV lung cancer and pneumonia	ND	
Gargar JD, *et al*.	2019	ND	Septic shock from ischemic colitis	ND	
Hubiche T, *et al*.	2019	5 months	Congenital heart disease, malnourishment, recurrent respiratory tract infections, repeated hospital/ICU admissions, and broad-spectrum antibiotics	Vancomycin	Susceptibility to vancomycin and penicillin (E-test)
Princess, *et al*.	2020	Middle aged	Type 2 diabetes, decompressive craniotomy, and broad-spectrum antibiotics	Teicoplanin	Susceptibility to ciprofloxacin and vancomycin. Resistance to penicillin (E-test)

*Bacillus clausii* safety profile has been confirmed in randomized control trials for efficient treatment of acute diarrhea related to viral infection, *Clostridioides difficile* infection, and antibiotic-associated diarrhea (^16^). Notwithstanding the published recommendations, the risks of *Bacillus* probiotics include the production of enteric toxins and toxicity for normal cells (^17^). Physicians are more aware of probiotics' benefits than of their risks, and, therefore, further research and case reporting are necessary to identify their potential harm in certain clinical contexts. We observed that Enterogermina™ was not efficient to treat our patient's diarrhea by enteropathogenic and enteroaggregative *E. coli* as diarrhea persisted for 11 days after its administration. Enterogermina™ (*Bacillus clausii*) administration in patients with recent upper gastrointestinal bleeding, acute diarrhea, and the use of proton pump inhibitors, which can diminish stomach pH and favor bacterial colonization, may lead to bacteremia through direct invasion or gut translocation. Therefore, *B. claussi* should be considered as a possible agent in the presence of systemic signs of infection, metabolic compromise, and hemodynamic instability when no other pathogens are identified to explain the clinical course. In such cases, the treatment should be aimed at fighting this Gram-positive rod with antibiotics other than clindamycin, erythromycin, and chloramphenicol, as *B. clausii* has intrinsic resistance against them (^4^).
